# Tubulin C-terminal Post-translational Modifications Do Not Occur in Wood Forming Tissue of *Populus*

**DOI:** 10.3389/fpls.2016.01493

**Published:** 2016-10-13

**Authors:** Hao Hu, Xi Gu, Liang-Jiao Xue, Prashant S. Swamy, Scott A. Harding, Chung-Jui Tsai

**Affiliations:** ^1^Daniel B. Warnell School of Forestry and Natural Resources, University of GeorgiaAthens, GA, USA; ^2^Department of Genetics, University of GeorgiaAthens, GA, USA; ^3^Institute of Bioinformatics, University of GeorgiaAthens, GA, USA

**Keywords:** detyrosination, non-tyrosination, glutamylation, acetylation, tension wood, microtubules

## Abstract

Cortical microtubules (MTs) are evolutionarily conserved cytoskeletal components with specialized roles in plants, including regulation of cell wall biogenesis. MT functions and dynamics are dictated by the composition of their monomeric subunits, α- (TUA) and β-tubulins (TUB), which in animals and protists are subject to both transcriptional regulation and post-translational modifications (PTM). While spatiotemporal regulation of tubulin gene expression has been reported in plants, whether and to what extent tubulin PTMs occur in these species remain poorly understood. We chose the woody perennial *Populus* for investigation of tubulin PTMs in this study, with a particular focus on developing xylem where high tubulin transcript levels support MT-dependent secondary cell wall deposition. Mass spectrometry and immunodetection concurred that detyrosination, non-tyrosination and glutamylation were essentially absent in tubulins isolated from wood-forming tissues of *P. deltoides* and *P. tremula* ×*alba*. Label-free quantification of tubulin isotypes and RNA-Seq estimation of tubulin transcript abundance were largely consistent with transcriptional regulation. However, two TUB isotypes were detected at noticeably lower levels than expected based on RNA-Seq transcript abundance in both *Populus* species. These findings led us to conclude that MT composition during wood formation depends exclusively on transcriptional and, to a lesser extent, translational regulation of tubulin isotypes.

## Introduction

Microtubules (MTs) are filamentous cytoskeleton components made up of α- and β-tubulins. In plants, MTs play critical roles in regulating intracellular trafficking, morphogenesis, and cellulose microfibril deposition during cell wall formation (Wasteneys, 2002). In the woody perennial *Populus*, α- (TUAs) and β-tubulins (TUBs) are encoded by relatively large multi-gene families, with highly conserved amino acid sequences (88–98% identities), except for the hypervariable C-terminus. The C-terminal tails of tubulin are of interest because they are hotspots for post-translational modifications (PTMs) in animals and protists (MacRae, 1997; Westermann and Weber, 2003; Magiera and Janke, 2014). Although tubulin gene expression (Oakley et al., 2007) and transgenic manipulation (Swamy et al., 2015) have been reported in *Populus*, characterization at the protein level has been largely unexplored.

Detyrosination, polyglutamylation and polyglycylation comprise the most well-characterized C-terminal PTMs of animal tubulins, in addition to acetylation that occurs in the N-terminus (Magiera and Janke, 2014). Immunological evidence for most of these PTMs has been reported in plants: detyrosination in tobacco, maize, grapevine and soybean; non-tyrosination (Δ2) in tobacco; polyglutamylation in tobacco, maize and soybean; and acetylation in numerous angiosperms (Smertenko et al., 1997, 1998; Huang and Lloyd, 1999; Wang et al., 2004; Parrotta et al., 2009; Jovanović et al., 2010; Nakagawa et al., 2013; Gzyl et al., 2015; Hotta et al., 2016). However, immunological detection can lead to equivocal conclusions (Hotta et al., 2016), especially when using animal-derived tubulin PTM antibodies of unknown specificities against plant tubulins (Parrotta et al., 2009). By comparison, mass spectrometry (MS)-based proteomics analysis provides a higher resolution approach for identification and quantification of tubulin isotypes and PTM isoforms (Verdier-Pinard et al., 2009; Miller et al., 2010). For clarity throughout, ‘isotype’ refers to genetically encoded tubulins and ‘isoform’ refers to their PTM variants (Parrotta et al., 2014). MS-based analysis is imperative for substantiating findings from antibody-based results, which can be equivocal due to high levels of sequence homology among numerous tubulin isotypes and the variable nature of (some) PTMs. For instance, detyrosinated and polyglutamylated tubulin isoforms were detected in tobacco suspension cells by immunofluorescence microscopy and immunoblotting (Smertenko et al., 1997), but were deemed absent in a recent study, also with cultured tobacco cells, by immunoblot and MS analyses (Hotta et al., 2016). The absence of detyrosination and polyglutamylation signals was also reported for *Arabidopsis* cells in the latter study (Hotta et al., 2016), challenging the occurrence of tubulin C-terminal PTMs in plants.

In our initial characterization of the *Populus* tubulin families, we described several *Populus* TUA genes that are predicted to harbor an unusual C-terminal Met, Glu or Gln instead of the evolutionarily conserved C-terminal Tyr (Oakley et al., 2007). This finding, along with discussion therein about the lack of an apparent homolog of tubulin Tyr ligase (TTL) in sequenced plant genomes (Oakley et al., 2007), raised the question whether the TUA detyrosination-tyrosination cycle is active in plants. Recently, we showed that detyrosination and non-tyrosination of TUA were negligible in *P. tremula* ×*alba* based on immunoblotting and MS analysis (Swamy et al., 2015). In the present study, we expanded the investigation to survey tubulin isotypes and their PTMs in *P. deltoides.* We focused on developing xylem because it undergoes extensive MT-dependent secondary cell wall thickening (Funada, 2008) and exhibits very high tubulin transcript levels especially in tension wood (TW; Oakley et al., 2007). TW fibers formed in response to gravitational stimuli are characterized by a cellulose-enriched gelatinous layer with increased MT abundance compared to normal wood (NW) fibers (Plomion et al., 2001; Pilate et al., 2004). While tubulin transcript levels increased substantially during TW formation (Oakley et al., 2007), there are no reports on whether auxiliary mechanisms including PTM may also become engaged. We took advantage of the proteomics dataset from *P. tremula* ×*alba* xylem (Swamy et al., 2015) for comparative analysis. In both cases, RNA-Seq-based *de novo* tubulin transcript assembly was undertaken in order to correct for sequence variations from the *P. trichocarpa* reference genome that could affect the accuracy of proteomics data analysis. The RNA-Seq data also permitted an assessment of xylem tubulin transcript abundance in both species. Our results indicated that C-terminal tubulin PTMs were undetectable in *Populus* xylem, in contrast to animal systems where their occurrence is commonplace. We interpret the results to suggest that genetically encoded diversity and other regulatory mechanisms supplant PTM modulation in *Populus*, even in MT-rich wood forming tissues.

## Materials and Methods

### Plant Materials

Bulk samples of developing xylem were scraped into liquid nitrogen from the debarked trunks of 5-year-old, field-grown *P. deltoides* trees. TW xylem was obtained from the upper side of the trunk leaned at a 30–40° angle from the vertical axis for 4 weeks. Snap-frozen samples were stored at -80°C until use.

### Tubulin Purification

Approximately 5 g of xylem tissue was ground into a fine powder in liquid nitrogen for tubulin purification using a modified DEAE-Sephadex chromatography method (Morejohn and Fosket, 1982; Smertenko et al., 1997). The tissue powder was suspended in 10 ml of PEM buffer (50 mM PIPES, pH 6.9; 0.5 mM MgCl_2_; 1 mM EGTA and 1 mM DTT) with protease inhibitors (1 mM benzamidine HCl; 2 mM leupeptin; 15 mM pepstatin A; 1 mM phenylmethylsulfonyl fluoride; 1 mM sodium fluoride and 50 μM N-tosyl-L-phenylalanine chloromethyl ketone) and 2 mM GTP, and vortexed vigorously. The mixture was first clarified at 50,000 g for 10 min, and the supernatant ultracentrifuged at 100,000 g for 45 min, both at 2°C. The resulting supernatant was mixed with 0.5 volumes of PEM-equilibrated DEAE-Sephadex A50 containing 0.5 mM GTP and incubated at 4°C for 1 h with gentle agitation. The mixture was loaded into a polyprep chromatography column (0.8 cm × 4 cm, BioRad), and washed with 3–5 volumes of 0.4 M KCl in PEM buffer containing 0.1 mM GTP. The bound tubulin proteins were then eluted with 0.8 M KCl in PEM buffer with 0.1 mM GTP. The protein-rich fractions were pooled and dialyzed against 1 L of 10 mM NH_4_HCO_3_ at 4°C overnight with one buffer change. The protein was concentrated using a Nanosep centrifugal column (MWCO 10K, Amicon) and the concentration was estimated using Bradford reagents (BioRad). Recombinant TUA1 in pET30a (Oakley et al., 2007) expressed in *E. coli* strain BL21 (DE3) was purified from inclusion body using BugBuster protein extraction reagent (Novagen), and quantified with BCA protein assay kit (Novagen).

### Western blotting

Seventy-five nanograms of purified tubulins were resolved on a 10% SDS-PAGE gel, and transferred onto an Immobilon-FL polyvinylidenedifluoride membrane (EMD Millipore). The membranes were incubated with the Blocking Buffer for Fluorescent Western Blotting (Rockland Immunochemicals) in phosphate-buffered saline, and then with primary and secondary antibodies in the same blocker containing 0.1% Tween 20. The following primary antibodies were used. Polyclonal antibodies (Open Biosystems) raised against recombinant TUA1 in rabbits (1:5000) and recombinant TUB15 in chickens (1:5000) recognize TUA and TUB proteins, respectively. Polyclonal rabbit antibodies raised against synthetic C-terminal peptides of TUA1 (anti-dY, ESPDGEDGDEGDE at 1:1000; and anti-dEY, ESPDGEDGDEGD at 1:1000; Sigma Genosys) recognize detyrosinated and non-tyrosinated TUA isoforms as described previously (Swamy et al., 2015). Mouse-derived monoclonal antibodies for polyglutamylated (clone B3, 1:1500) and acetylated (clone 6-11B-1, 1:500) tubulins were obtained from Sigma. Hybridization signals were detected using IRDye 680RD-conjugated goat anti-rabbit IgG, 800CW-conjugated goat anti-mouse or 800CW-conjugated donkey anti-chicken secondary antibodies (1:15000, Li-cor) with an Odyssey infrared imaging system (Li-cor).

### Mass Spectrometric Analysis

The SDS-PAGE bands containing tubulins were excised, destained and subjected to in-gel trypsin digestion as described (Shevchenko et al., 1996) at the proteomics core of Michigan State University. Peptides were fractionated by reverse phase HPLC using a Waters nanoAcquity UPLC. Eluted peptides were sprayed into a ThermoFisher LTQ Linear Ion trap mass spectrometer outfitted with a MICHROM Bioresources ADVANCE nano-spray source. The top five ions in each survey scan are then subjected to data-dependent zoom scans followed by low energy collision induced dissociation (CID) and the resulting MS/MS spectra are converted to peak lists in BioWorks Browser v3.2 using the default LTQ instrument parameters. Peak lists were searched against all *Populus* tubulin sequences using the Mascot searching algorithm v2.1. The Mascot output was then analyzed using Scaffold, v1.7.0 to probabilistically validate protein identifications.

An independent set of purified tubulin samples was subjected to SDS-PAGE and in-gel CNBr digestion according to Xiao et al. (2010) at the Proteomics core of the Albert Einstein College of Medicine. Peptides were fractionated by reverse phase HPLC (Ultimate 3000, Dionex), followed by MALDI-TOF analysis (ABI 4800, Applied Biosystems) in both positive and negative ion mode as detailed in (Xiao et al., 2010). *Populus* tubulin sequences were used as the database. Data were processed with Data Explorer software (v4.9). Label-free quantitation of tubulin isotypes was performed according to Miller et al. (2012).

### RNA-Seq Analysis

The total RNA was extracted with the Direct-zol RNA Kit (Zymo Research) using Plant RNA Reagent (Life Technologies) and quantified with the Qubit RNA HS Assay Kit on a Qubit fluorometer (Life Technologies). RNA-Seq libraries were prepared using the Illumina TruSeq Stranded RNA LT Kit and sequenced on an Illumina NextSeq 500 at the Georgia Genomics Facility of University of Georgia. The data are available at NCBI Sequence Read Archive under accession number SRP076604. After filtering to remove rRNA sequences, data were processed by a local assembly pipeline (Gu and Tsai, unpublished) modified from Allen et al. (2015) for *de novo* assembly of *TUA* and *TUB* sequences. Briefly, *TUA* and *TUB* transcript sequences from *P. trichocarpa* were used as reference for read mapping by Bowtie 2, v2.2.3 (Langmead and Salzberg, 2012). Matched reads were pooled and subjected to *de novo* assembly by Trinity (Haas et al., 2013). The assembled contigs were Blasted against the reference, and relevant sequences were retained as new baits to repeat the process until the output was stable, usually within 10 iterations depending on transcript abundance. Following manual curation, the longest contig for each gene was retained for further analysis. This procedure was also performed for a previously published *P. tremula* ×*alba* xylem RNA-Seq dataset (SRP042117; Swamy et al., 2015). The translated peptide sequences were used to calculate the theoretical *m/z* for peptide mapping. For expression analysis, a region of up to 200 bp covering the hypervariable C-terminus and a portion of the 3′-UTR was retrieved for each gene from available transcript assemblies of *P. deltoides* and *P. tremula* ×*alba* above, guided by multiple sequence alignment. The *P. trichocarpa* sequences were used for those that were absent in our assemblies (not expressed). Reads were mapped to the reference sequences using Blat and filtered by >95% hit length coverage with a 2% mismatch allowance. Reads were only assigned to the best hit. Transcript abundance was estimated by fragments per million total reads per kilobase (FPKM).

## Results

### Mass Spectrometric Analysis of Tubulin Abundance in Poplar Xylem

**FIGURE 1:**
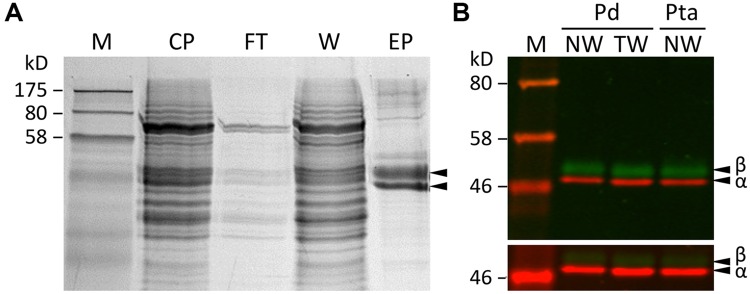
**Tubulin purification. (A)** SDS-PAGE analysis of xylem protein extracts from various steps of the tubulin purification process. M, markers; CP, crude proteins; FT, flow-through; W, washed, unbound proteins; EP, eluted proteins enriched with tubulins. **(B)** A two-color Western blot of purified tubulins from *P. deltoides* (Pd) and *P. tremula* ×*alba* (Pta) normal wood (NW) or tension wood (TW) xylem probed with anti-TUA (red channel) or anti-TUB (green channel) antibodies. The scanning intensities were adjusted in the upper panel (reduced for red and increased for green) in order to visualize both TUA and TUB signals clearly. The lower panel was scanned at the same intensity for both channels.

Tubulin proteins were purified from developing xylem of *P. deltoides* using a modified DEAE-Sephadex anion exchange chromatography protocol (Swamy et al., 2015), originally developed for plant cell suspension cultures (Morejohn and Fosket, 1982). Based on SDS-PAGE and Coomassie Brilliant Blue staining, the purified proteins were enriched in a doublet of ∼50 kDa, close to the expected size for tubulins (**Figure 1A**). Western blotting confirmed that the lower band was TUA, and the upper one was TUB (**Figure 1B**). The yield of tubulins was 40–60 μg/g fresh-weight of developing xylem. The excised tubulin bands were subjected to in-gel cyanogen bromide (CNBr) digestion for proteomics analysis by MS.

**FIGURE 2:**
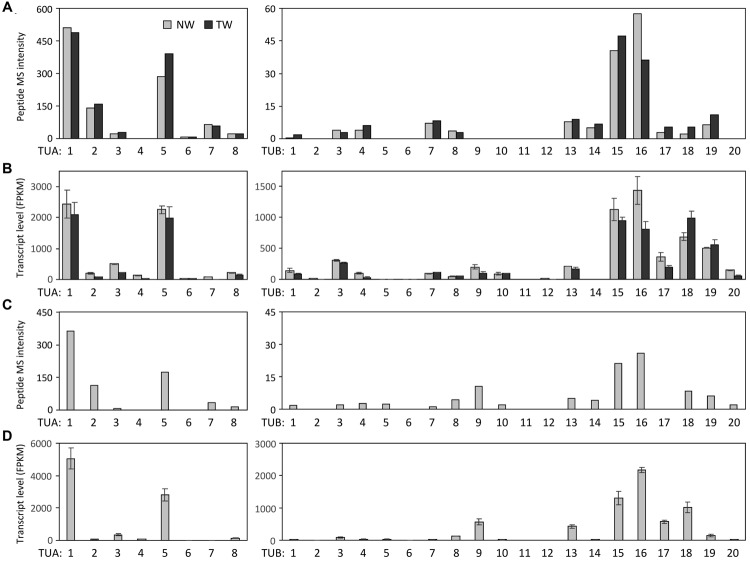
**Tubulin isotype and transcript abundance in xylem. (A)** Label-free MS quantification of TUA (left panel) and TUB (right panel) reporter peptides in normal wood (NW) and tension wood (TW) of *P. deltoides*. **(B)** *TUA* and *TUB* transcript abundance based on RNA-Seq analysis of *P. deltoides* NW and TW samples (*n* = 2). **(C)** Label-free MS quantification of TUA and TUB based on a previously published *P. tremula* ×*alba* NW xylem dataset (Swamy et al., 2015). **(D)** *TUA* and *TUB* transcript abundance based on RNA-Seq analysis of *P. tremula* ×*alba* NW samples (*n* = 3).

A label-free method (Miller et al., 2012) was applied to determine the relative abundance of tubulin isotypes in NW and TW samples of *P. deltoides* using isotype-specific C-terminal reporter peptides (**Table 1**). RNA-Seq data from the same series of samples were used for *de novo* assembly of (expressed) *P. deltoides* tubulin transcripts in order to correct for sequence polymorphisms that deviate from the *P. trichocarpa* reference gene models (see “Materials and Methods” and **Table 1**). TUA1 and TUA4/5 were the most abundant isotypes in both NW and TW (**Figure 2A**). The predicted *m/z* for the TUA4 and TUA5 reporter peptides is indistinguishable by MS (**Table 1**). However, as *TUA4* transcripts were barely detected in xylem based on RNA-Seq analysis (**Figure 2B**), the TUA4/5 reporter peptide signal probably represents (and hence was assigned to) TUA5 (**Figure 2A**). The TUB signals were much lower than the TUA signals, consistent with the immunoblot results shown in **Figure 1**. TUB15 and TUB 16 were the predominant TUB isotypes in *P. deltoides* xylem. The MS abundances of TUA and TUB isotypes were largely similar between NW and TW samples of *P. deltoides* (**Figure 2A**). We also analyzed the proteomics dataset of wild-type (WT) *P. tremula* ×*alba* xylem (NW) described in Swamy et al. (2015), with the same RNA-Seq-based sequence curation as above (**Figure 2C**). The TUA and TUB isotype profiles were generally consistent between species, with the exception of TUB9 which was among the top three most abundant C-terminal TUB peptides in *P. tremula* ×*alba* xylem (**Figure 2C**), but was undetected in *P. deltoides* (**Figure 2A**).

**Table 1:** Reporter peptides used in label-free quantification of TUA and TUB isotypes.

Isotype	Residues	*P. deltoides*	*m/z*	*P. tremula × alba*	*m/z*
TUA1	414–451	EEGEFSEAREDLAALEKDYEEVGAESPDGEDGDEGDEY	4180.691	EEGEFSEAREDLAALEKDYEEVGAESPDGEDGDEGDEY	4180.691
TUA2	414–450	EEGEFSEAREDLAALEKDYEEVGAEGVDDEEDNEDYE	4224.717	EEGEFSEAREDLAALEKDYEEVGAEGVDDEEDNEDYE	4224.717
TUA3^a^	414–451	EEGEFSEAREDLAALEKDYEEVGAETAEGDDEEGEEYM^∗^	4222.738	EEGEFSEAREDLAALEKDYEEVGAESAEGDDEDGEEYM^∗^	4194.707
TUA4	414–450	EEGEFSEAREDLAALEKDYEEVGAEGVDDEEEGDDYQ	4166.712	EEGEFSEAREDLAALEKDYEEVGAEGVDDEEEGDDYQ	4166.712
TUA5	414–451	EEGEFSEAREDLAALEKDYEEVGAESAEGDDDDGDEYM^∗^	4166.675	EEGEFSEAREDLAALEKDYEEVGAESAEGDDDDGDEYM^∗^	4166.675
TUA6	414–449	EEGEFSEAREDLAALEKDYEEVGAEGGDEEGEEEDY	4024.637	EEGEFSEAREDLAALEKDYEEVGAEGGDEEGEEEDY	4024.637
TUA7^a^	414–451	EEGEFSEAREDLAALEKDYEEVGAESAEGEDDDGEEYM^∗^	4194.707	EEGEFSEAREDLAALEKDYEEVGAESAEGEDDEGEEYM^∗^	4208.722
TUA8	414–449	EEGEFSEAREDLAALEKDYEEVGAEGGDDEGEDEDY	3996.606	EEGEFSEAREDLAALEKDYEEVGAEGGDDEGEDEDY	3996.606
TUB1	416–449	NDLVSEYQQYQDATADEEGEYEDEEEGEYQGDYQ	4038.559	NDLVSEYQQYQDATADEEGEYEDEEEGEYQGDYQ	4038.559
TUB2^a^	416–450	NDLVSEYQQYQDATADEEGEYEEEEEGEEYQQDYQ	4252.655	NDLVSEYQQYQDATADEEGEYEEEEEGDEYQQDYQ	4238.630
TUB3	416–447	NDLVSEYQQYQDATADEEGEFEDEEEAYGDEA	3688.400	NDLVSEYQQYQDATADEEGEFEDEEEAYGDEA	3688.400
TUB4	416–446	NDLVSEYQQYQDATADEEGEYEDEEAYQDED	3690.400	NDLVSEYQQYQDATADEEGEYEDEEAYQDED	3690.400
TUB5	416–444	NDLVSEYQQYQDATADEDYEDEEEELHDM^∗^	3475.373	NDLVSEYQQYQDATADEDYEDEEEELHDM^∗^	3475.373
TUB6^b^	416–443	NDLVSEYQQYQDATTYEDCEDEEELHDM^∗^	3364.320	NDLVSEYQQYQDATTYEDCEDEEELHDM^∗^	3364.320
TUB7^b^	416–445	NDLVAEYQQYQDATADDEEYEEEEEEEIGA	3524.414	NDLVAEYQQYQDATADDEEYEEEEEEEIGA	3524.414
TUB8	416–445	NDLVAEYQQYQDATIDEEEYEEEEEEEHDT	3692.500	NDLVAEYQQYQDATIDEEEYEEEEEEEHDT	3692.500
TUB9^a^	416–442	NDLVSEYQQYQDAVADNEGEYDEEEPM^∗^	3132.271	NDLVSEYQQYQDAAADNEGEYDEEEPM^∗^	3104.240
TUB10^a^	416–444	NDLVSEYQQYQDAAADNDDEYDEEEIVEN	3423.378	NDLVSEYQQYQDAAADNDDEYDEEEAM^∗^ (442)	3122.214
TUB11^b^	416–444	NDLVAEYQQYQDATAEEEIEYEEDDGVEN	3408.403	NDLVAEYQQYQDATAEEEIEYEEDDGVEN	3408.403
TUB12^b^	416–444	NDLVAEYQQYQDATTEEDIEYEEEDGVEN	3438.414	NDLVAEYQQYQDATTEEDIEYEEEDGVEN	3438.414
TUB13	411–442	NDLVSEYQQYQDATAEDDIDYEDEEEEEAAEM^∗^	3738.473	NDLVSEYQQYQDATAEDDIDYEDEEEEEAAEM^∗^	3738.473
TUB14^b^	416–446	NDLVSEYQQYQDATADEEVDYEDEEEEEAEM^∗^	3667.436	NDLVSEYQQYQDATADEEVDYEDEEEEEAEM^∗^	3667.436
TUB15	416–445	NDLVSEYQQYQDATVDEELEYEDEEEEEAA	3582.456	NDLVSEYQQYQDATVDEELEYEDEEEEEAA	3582.456
TUB16^a^	416–446	NDLVSEYQQYQDATADEEVDYEDEEEDAAGM^∗^	3523.390	NDLVSEYQQYQDATAEEEVDYEDEEEDAAGM^∗^	3537.410
TUB17	416–448	NDLVSEYQQYQDATADEEGEYEDEEDGQYAEQM^∗^	3843.506	NDLVSEYQQYQDATADEEGEYEDEEDGQYAEQM^∗^	3843.506
TUB18	416–450	NDLVSEYQQYQDATADEEGEYDDEEEEEGQYAEQM^∗^	4101.591	NDLVSEYQQYQDATADEEGEYDDEEEEEGQYAE (448)	3890.496
TUB19	419–449	NDLVAEYQQYQDATIEEDGEYEEEGEENYDA	3658.462	NDLVAEYQQYQDATIEEDGEYEEEGEENYDA	3658.462
TUB20^a^	419–449	NDLVAEYQQYQDATVEEDGEYEEEGEENYDD	3688.400	NDLVAEYQQYQDATVEEDGEYEVEGEENYDD	3658.462

*^a^Sequences deviating from the P. trichocarpa reference are shown in red. ^b^sequences in gray were not recovered from de novo transcript assembly; instead, the P. trichocarpa reference sequences were used. ^∗^modification of Met to homoserine lactone during CNBr reaction.*

### Detection of Tubulin PTMs

**FIGURE 3:**
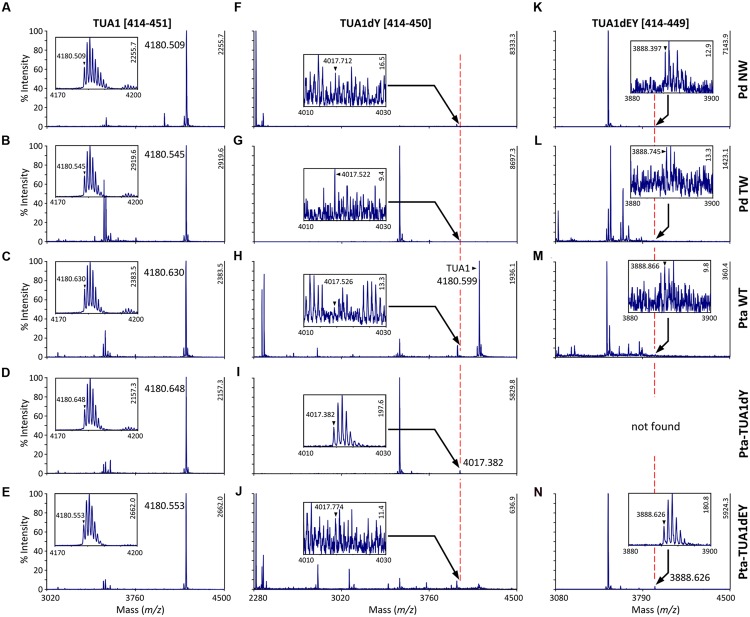
**MS analysis of TUA detyrosination and non-tyrosination.** Mass spectra of the C-terminal CNBr peptides of TUA1 **(A–E)**, its detyrosinated (TUA1dY, **F–J**) or non-tyrosinated (TUA1dEY, **K–N**) isoforms. The primary axis is scaled to the most abundant peptide in each panel, with intensity on the secondary axis. Insets show mass spectra of the respective C-terminal peptide. The monoisotopic mass is denoted by a triangle. Insets in **(A–E,I,N)** show consistent isotopic profiles. The peptide identities in **(C,I,N)** were previously confirmed by MALDI-TOF/TOF (Swamy et al., 2015). Peptide signals in all other insets **(F–H,J–M)** lacked clear isotopic distribution and are deemed background noise.

**FIGURE 4:**
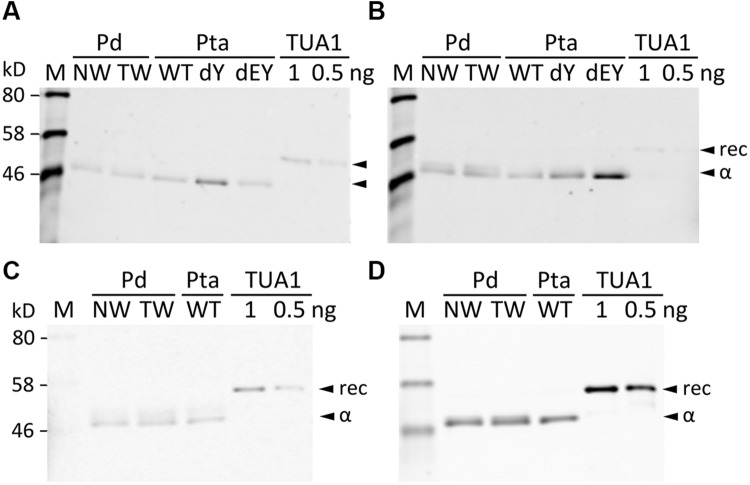
**Western blotting with PTM antibodies.** Blots were probed with anti-TUA1dY **(A)**, anti-TUA1dEY **(B)**, anti-glutamylation **(C)** or anti-TUA **(D)** antibodies. Tubulin-enriched extracts from NW and TW of *P. deltoides* (Pd), NW of *P. tremula* ×*alba* (Pta) WT and transgenic (dY or dEY) plants, or recombinant TUA1 (rec) were used. Specific signals for detyrosinated and non-tyrosinated TUA1 were only detected in transgenic dY and dEY plant extracts, respectively. Signals from the anti-glutamylation antibody were seen for the recombinant TUA1 and were deemed non-specific.

We searched the *P. deltoides* MS spectra of C-terminal polypeptides for evidence of tubulin detyrosination, non-tyrosination or glutamylation. The tyrosinated (unmodified) C-terminal peptide (residues 414–451) of TUA1 was detected with an *m/z* of 4180.5, very near the theoretical monoisotopic mass (MH+) of 4180.69 (**Figures 3A,B**). Its authenticity was previously confirmed by MALDI-TOF/TOF analysis using enriched tubulins purified from NW xylem of WT *P. tremula* ×*alba* (Swamy et al., 2015) (**Figure 3C**). In that same study, we also identified and MALDI-TOF/TOF-confirmed C-terminal peptides of detyrosinated (dY, *m/z* 4017.4) and non-tyrosinatable (dEY, *m/z* 3888.6) isoforms from transgenic *P. tremula* ×*alba* ectopically expressing the dY and dEY PTM mimics of TUA1, respectively (Swamy et al., 2015) (**Figures 3I,N**). However, the MS signal for either PTM peptide was near background in *P. deltoides* NW and TW samples or in WT *P. tremula* ×*alba* (**Figure 3F–H,K–M**) (Swamy et al., 2015). This was corroborated by Western blot analysis of purified xylem tubulins from both *Populus* species using PTM-specific antibodies raised against the dY (anti-dY) or dEY (anti-dEY) C-terminal peptide of TUA1. As reported previously (Swamy et al., 2015), the anti-dY and anti-dEY immunosignals were clearly detected with xylem tubulins purified from dY- and dEY-expressing transgenics, respectively (**Figures 4A,B**). The faint signals observed in the other samples likely represent background hybridization as seen for the recombinant TUA1 protein purified from *Escherichia coli* that was included as a negative control (**Figures 4A,B**). The results suggest that detyrosination and non-tyrosination of TUA were either absent or negligible in *Populus* xylem.

Polyglutamylation of *Populus* tubulins was examined by immunoblotting using a commercial monoclonal antibody raised against polyglutamylated TUAs of sea urchin (*Lytechinus pictus*) (Gagnon et al., 1996). A weak signal that overlapped with the anti-TUA signal was detected for all samples, including recombinant TUA1, indicative of background hybridization likely due to the TUA origin of the monoclonal antibody (**Figures 4C,D**). We then searched the MALDI mass spectra for peptide masses that were 129 Da (the mass of a Glu residue) larger than the C-terminal peptide masses of TUAs and TUBs in both *Populus* species. No such signals were found above background levels, suggesting that tubulin glutamylation is unlikely to occur in wood-forming tissues of *Populus*.

**FIGURE 5:**
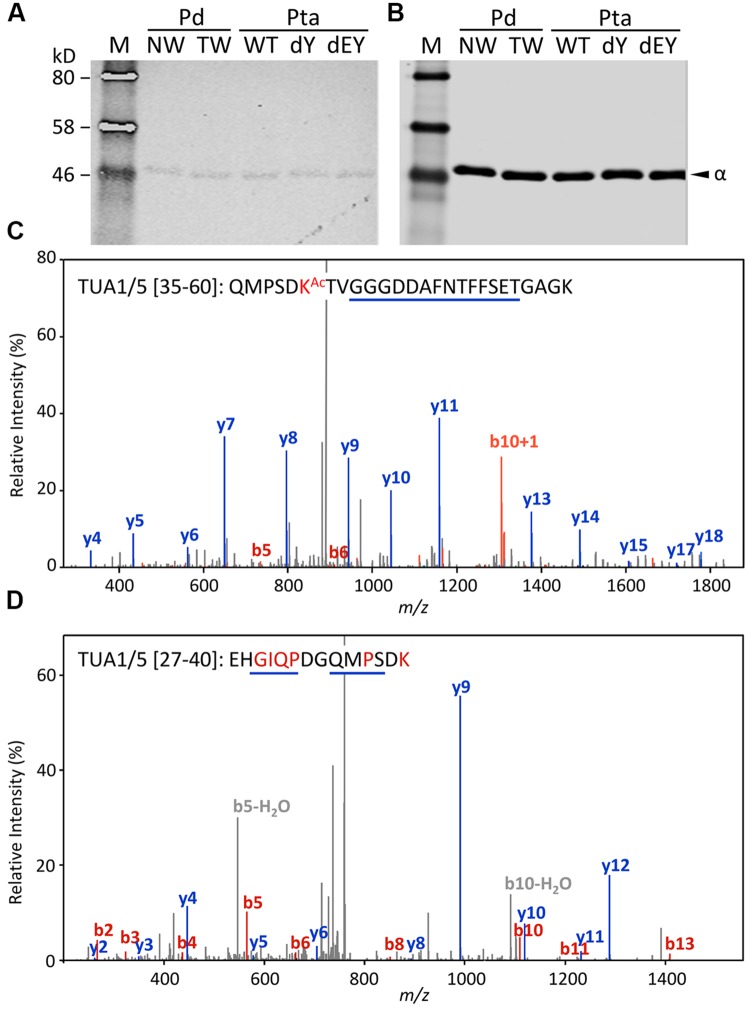
**Acetylation of TUA. (A,B)** The enriched tubulins were probed with anti-acetylated TUA **(A)** or anti-TUA **(B)** antibodies. Samples are the same as in **Figure 4**. **(C)** MS/MS spectra of a trypsin peptide shared by TUA1 and TUA5. The mass difference between b5 and b6 ions corresponds to an acetylated Lys residue. **(D)** MS/MS spectra of a trypsin peptide cleaved at Lys-40, shared by unmodified TUA1 and TUA5. The b ions and y ions are labeled in red and blue, respectively.

Acetylation of TUAs was studied using a commercial monoclonal antibody raised against acetylated TUA of sea urchin. As with the other immunoblot analyses described above, only background hybridization signals were detected regardless of tissue (NW or TW) or genotype (**Figures 5A,B**). A shot-gun LC-MS/MS analysis of trypsin-digested tubulin identified an N-terminal peptide shared by TUA1 and TUA5 that appeared to be acetylated at Lys-40 due to a mass shift of 42 Da (**Figure 5C**). Although this peptide was identified with a 94% probability, the acetylated Lys-40 signals (b5 and b6 ions) were very low. Unmodified Lys-40 is susceptible to trypsin cleavage, and accordingly, we identified a shorter N-terminal TUA1/TUA5 peptide with an exposed Lys-40 (**Figure 5D**). As TUA1 and TUA5 are abundant in *Populus* xylem, we interpret the immunoblot and MS data to suggest that both isotypes were subjected to very low levels of acetylation at the conserved Lys-40 residue.

### RNA-Seq Analysis of TUA and TUB

The RNA-Seq data were also processed to estimate tubulin transcript abundance in xylem of both *P. deltoides* and *P. tremula* ×*alba*. Because *TUA* and *TUB* genes share high degrees of sequence identity, we extracted up to 200 bp sequences spanning the hypervariable C-termini and 3′-UTRs from the *de novo* transcript assemblies as references for read mapping (see “Materials and Methods” and Supplementary Data 1). This was intended to minimize ambiguous read alignment in the highly conserved coding region. The overall *TUA* and *TUB* transcript profiles were similar between *P. deltoides* and *P. tremula* ×*alba*, suggesting a conserved transcriptional regulation in this genus (**Figures 2B,D**). The transcript abundance estimates by RNA-Seq were largely congruent with the isotype profiles observed in both species (**Figure 2**), suggesting transcriptional regulation. Exceptions to this general trend were two TUBs with discordant patterns. Low MS signals were detected for TUB18 despite its very high transcript levels in both species, and TUB17 exhibited a similar though less pronounced pattern. The data suggested involvement of post-transcriptional or translational regulation. As mentioned above, the TUB9 reporter peptide was detected in hybrid aspen *P. tremula* ×*alba*, but not *P. deltoides* (**Figures 2A,C**). Accordingly, *TUB9* transcripts were present at relatively higher levels in *P. tremula* ×*alba* (75th percentile of all *TUBs*) than in *P. deltoides* (50–55th percentile) xylem (**Figures 2B,D**). The results hint at a taxon-specific fine tuning of *TUB* regulation in *Populus*.

The proteomics data also help curate gene model annotation of the reference genome. We previously cloned all eight TUA cDNAs from *P. tremuloides* (Oakley et al., 2007). The experimentally identified *TUA5* mRNA (GenBank accession EF583814) differed from the primary gene model (Potri.009G085100.1) predicted in the current *P. trichocarpa* genome v3 (Phytozome v11), but matched the secondary gene model (Potri.009G085100.2). The two models represent splice variants, with the primary gene model predicted to harbor an extra, 31-bp intron upstream of the stop codon that is not found in other Class I TUAs (Oakley et al., 2007). Proteomics data supported the presence of Potri.009G085100.2 in both *P. deltoides* (NW and TW) and *P. tremula* ×*alba* (NW) xylem, and no signal matching the predicted Potri.009G085100.1 C-terminal peptide was detected. Moreover, *de novo* assembly of xylem RNA-Seq data from both *P. deltoides* (this study) and *P. tremula* ×*alba* (Swamy et al., 2015) using a custom local assembly pipeline (Gu and Tsai, unpublished) recovered only the Potri.009G085100.2 transcript (Supplementary Data 2). Taken together, both transcriptomics and proteomics data suggest that Potri.009G085100.2 is the predominant TUA5 isotype in *Populus*, and the splice variant Potri.009G085100.1 is likely an annotation artifact.

## Discussion

*Populus* possesses a relatively large tubulin gene family composed of 8 *TUAs* and 20 *TUBs* (Oakley et al., 2007). Their transcripts exhibit differential tissue distribution, with several being strongly and preferentially expressed in developing xylem undergoing secondary cell wall thickening. In fact, *TUA1* and *TUA5* are among the top twenty most abundant transcripts in developing xylem of NW and TW based on RNA-Seq analysis of both *P. deltoides* and *P. tremula* ×*alba*. This is consistent with an important role of MTs in cellulose deposition, a plant-specific evolutionary innovation of MT function (Wasteneys, 2002). Historically, tubulin-rich brain tissue has been the *de facto* source for tubulin purification and PTM investigation in mammalian systems (Sackett et al., 2010). We reasoned that tubulin-rich xylem would be an ideal tissue for similar studies to investigate the role of tubulin PTMs, if any, during MT-dependent cell wall biogenesis.

The isotype abundance estimates from MALDI-TOF analysis were largely consistent with transcript profiling by RNA-Seq, with TUA1, TUA5, TUB15 and TUB16 being the predominant tubulin isotypes in *Populus* xylem. However, peptide signals were weak for a few highly expressed *TUBs* in both *Populus* species examined. This suggests that TUBs may also be targets of translational or other post-transcriptional regulation by as-yet-unidentified mechanisms. The abundance differentials between TUA and TUB previously observed at the transcript level (Oakley et al., 2007) were confirmed at the protein level in the present investigation. In fact, the abundance differentials between TUA and TUB were much greater at the protein level (∼10-fold) than the transcript level (∼2-fold) for the predominant isotypes (**Figure 2**), further supporting the idea of additional regulatory mechanisms acting on TUB. Previously, we showed that several xylem-expressed *TUA* and *TUB* genes were up-regulated in TW relative to NW of *P. tremuloides* (Oakley et al., 2007). This TW response, however, was not observed in the present study with *P. deltoides*, either at the transcript, isotype or PTM level (**Figures 2–5**). This discrepancy might be attributed to a shorter TW induction period (4 weeks) in the present experiment than that (>3 months) reported in Oakley et al. (2007). It could also reflect varying degrees of strain due to large (5-year-old, present study) versus small trees (2-year-old, previous work) in response to bending, although genetic or ontogenetic influences could not be excluded. Future work is needed to investigate the underlying differences in TW response. Despite the absence of treatment effects, tubulin proteins and transcripts remained highly enriched in both NW and TW xylem of *P. deltoides* (see **Figure 1** and discussion above). The TW sample thus served as an independent replicate for tubulin PTM assessment.

Lys-40 acetylation of TUA1/TUA5 was the only tubulin PTM confirmed by MS/MS in *Populus* xylem, although the immunosignals were negligible using a commercial monoclonal antibody that has been used to detected acetylated TUAs in a wide range of plant species (Huang and Lloyd, 1999; Tran et al., 2012; Nakagawa et al., 2013; Gzyl et al., 2015). Tubulin acetylation was detected at high levels in tobacco cell cultures but at much lower levels in *Arabidopsis* cell cultures (Hotta et al., 2016). Variable levels of tubulin acetylation were also reported in a broad survey of 15 angiosperm species (Nakagawa et al., 2013) or between different tissues (Wang et al., 2004). Although plants lack apparent homologs of the animal acetyltransferase MEC-17 that catalyzes TUA Lys-40 acetylation (Akella et al., 2010), *Arabidopsis* histone acetyltransferase ELP3 and histone deacetylase HDA14 have been shown to be highly enriched on MTs and may take part in TUA acetylation/deacetylation (Tran et al., 2012). Data mining of a multi-tissue (leaf, xylem, bark and root) RNA-Seq dataset (Xue et al., 2016) suggested that the *Populus* ELP3 and HDA14 orthologs were mainly expressed in leaves. Their transcripts were barely detected (FPKM ≤ 5) in developing xylem of greenhouse-grown *P. tremula* ×*alba* (Swamy et al., 2015; Xue et al., 2016). In field-grown *P. deltoides*, ELP3 orthologs were also poorly expressed, but deacetylase HDA14 orthologs were detected at higher levels (FPKM ∼15). Poor expression of acetyltransferases and deacetylases, or higher deacetylase than acetyltransferase transcript levels could both explain the low levels of TUA acetylation that we observed in NW and TW tissues.

None of the C-terminal PTMs frequently reported for animal tubulins, including detyrosination, non-tyrosination and glutamylation, was detected by MS or immunoblotting above background noise in *Populus* xylem. Low levels of tubulin C-terminal PTM immunosignals were previously reported for tobacco suspension cells (Smertenko et al., 1997), maize leaves, roots, pollen and anthers (Wang et al., 2004) and soybean seedlings (Gzyl et al., 2015). However, a recent study employing an improved (tubulin binding protein-based) affinity purification of tubulins from tobacco and *Arabidopsis* cell cultures showed no evidence of C-terminal PTMs by immunoblot or MS analyses (Hotta et al., 2016), similar to our observations in *Populus* xylem. It thus appears that tubulin C-terminal PTMs are absent or rare in plants unlike in animals or protists. What underlies this evolutionary distinction between plants and other eukaryotes remains unclear, but several considerations can be offered. First, reversible PTMs are thought to increase tubulin pool diversity, and hence the flexibility of MT function (Westermann and Weber, 2003). This diversity may be fulfilled by genetically encoded tubulins in *Populus* and other plant species that are more numerous and heterogeneous than their animal counterparts. As we reported previously, five of the eight *Populus* TUAs encode a unique C-terminal Met (TUA3, TUA5 and TUA7), Glu (TUA2) or Gln (TUA4), instead of the typical Tyr (TUA1, TUA6 and TUA8) necessary for participation in the detyrosination-tyrosination cycle (Oakley et al., 2007). MS detection of the C-terminal peptide of TUA1 (Y-type), TUA5 (M-type) and TUA2 (E-type) provided a convincing argument for a genetically diverse pool of TUA in poplar xylem, regardless of PTM activities.

Second, tubulin C-terminal tails exposed on the outer surface of MTs interact with MT-associated proteins (MAPs), and as such, their PTMs are thought to modulate MT–MAP interactions (Magiera and Janke, 2014). Analysis of sequenced genomes has shown that MAP families are generally more diverse in animals than in plants, both in number of families and number of genes within each family (Gardiner, 2013). The extent to which tubulin C-terminal PTMs parallel the complexity of MAP–MT interactions during evolution is an interesting possibility to consider. One exception to the MAP family diversity noted above is that the kinesin family has significantly expanded in plants (Lee and Liu, 2004). Kinesins and other motor proteins play important roles in self-organization of plant MTs in the absence of canonical centrosomes (Wasteneys, 2002). Plant kinesins have expanded functions from cell cycle to morphogenesis to signal transduction (Li et al., 2012). Thus, MT multiplexity in plants can also be fulfilled by mechanisms other than tubulin C-terminal PTMs.

Vertebrate tubulin PTMs are under strict spatiotemporal regulation (Westermann and Weber, 2003; Janke and Bulinski, 2011). Thus, another explanation for the lack of PTM signals in poplar xylem could be their conditional occurrence in certain tissues and cells, or during specific developmental stages that were not captured in the present investigation. In closing, we found no evidence of tubulin C-terminal PTMs in developing xylem of *Populus* undergoing extensive MT-dependent cell wall biogenesis. Our data suggest that transcriptional, post-transcriptional and/or translational regulation of a genetically more diverse tubulin repertoire features more prominently than tubulin PTMs during wood formation in *Populus.*

## Author Contributions

C-JT and SH designed the research, PS performed tubulin purification, HH performed Western blotting and analyzed proteomics data, XG and L-JX performed RNA-Seq analysis, HH, SH, and C-JT wrote the manuscript. All authors read and approved the manuscript.

## Conflict of Interest Statement

The authors declare that the research was conducted in the absence of any commercial or financial relationships that could be construed as a potential conflict of interest.
