# Supplementary material for: Tubulin C-terminal Post-translational Modifications Do Not Occur in Wood Forming Tissue of Populus
Source: Front Plant Sci. 2016 Oct 13;7:1493. doi: 10.3389/fpls.2016.01493 (PMC5061773; doi:10.3389/fpls.2016.01493)
Supplement: Supplementary file 1 [file Data_Sheet_1.DOC]

Supplementary Material

# Tubulin post-translational modifications occur at low levels in wood forming tissue of *Populus*

Hao Hu, Xi Gu, Liang-Jiao Xue, Prashant S. Swamy, Scott A. Harding, Chung-Jui Tsai1*

*** Correspondence:** Chung-Jui Tsai, [cjtsai@uga.edu](mailto:cjtsai@uga.edu)

# Supplementary Data

# Supplementary Data 1. Fasta sequences of the 3’ region obtained from de novo assemblies of *P. deltoides* (Pd) and *P. tremula* × *alba* (Pta) RNA-Seq data for transcript abundance estimation and for reporter peptide sequence curation. When unavailable, *P. trichocarpa* sequences were used (Potri gene models).

# Supplementary Data 2. Sequence alignment of the predicted *P. trichocarpa* TUA5 splice variants with the *P. deltoides* and *P. tremula* × *alba* transcripts obtained from de novo assembly. Predicted start and stop codons are shown in red.

**Supplementary Data 1.** Fasta sequences of the 3’ region obtained from *de novo* assemblies of *P. deltoides* (Pd) and *P. tremula* × *alba* (Pta) RNA-Seq data for transcript abundance estimation and for reporter peptide curation. When unavailable, *P. trichocarpa* gene model sequences (Potri) were used.

>PdTUA1

TGCCCTGGAGAAGGATTACGAGGAGGTTGGGGCTGAATCTCCTGATGGAGAGGATGGTGATGAAGGAGATGAGTACTGATAATAGGAAGTGTACTTCCTTTTGTTTTTTTGTTTGGCTGCTATGAGTTTGCTTAGACCATGTCTGTTGGTACGTGTGATCTTTGGTATTGTAATAAAAAAAATACAAGGATTGGAGCCCT

>PdTUA2

TGCCCTTGAGAAGGATTACGAGGAAGTTGGTGCTGAAGGTGTAGATGATGAAGAAGACAATGAAGATTACGAATAAGTAATTACCAGCCAGCAATCGAGAAGGACCGTGAGGGTTTGGCCACCAACCCTCCCCTTCTTGCTTCGCAGCTGTTATGTTTGTGATTTGTGGCACCGCTATCCTTCAGTAAAACAGTTAAAAA

>PdTUA3

TGCACTCGAGAAGGATTATGAAGAGGTGGGTGCGGAAACAGCCGAGGGCGATGATGAAGAGGGCGAGGAGTACATGTGAGGTGCCCGCATCACCTGTCTCTCTAATGAAGTGCCTGACAATTTGATACGAGTTGTTGTATGGCAGTGCCATGTGTCCTGTGTTTTTTATTTTTTCAGTTGAGAATTATTCATTTCAGGTA

>PdTUA4

TGCCCTTGAGAAGGATTACGAGGAAGTTGGTGCTGAAGGTGTAGATGATGAAGAGGAAGGTGATGACTATCAATAAGTTATTGCCACTTGCAATAGAATTCATCTGGGAAATGGTTAAGCGTGTGAGGGTTTGGCCACCAACCCGCCCGTCTTACGTTGCCGTTGTTATGTTTGTGATGTGTCGCGCCACTATCCTTCTT

>PdTUA5.2

CGCCCTTGAGAAGGATTATGAGGAGGTCGGTGCTGAATCAGCTGAGGGTGATGATGATGATGGTGACGAGTACATGTGAAGTGCTTCGTAGTGGGTGTAGCCCTAATATGATGTGTATCTGTATGGCATTGCCGTATGTTGTATTTGTTTTTTAATGAATATCTACATGTAATTGCACCATTTGAAGCTTTTATTGTTTC

>Potri.013G056800|TUA6

TGCTCTTGAGAAAGACTATGAGGAAGTCGGAGCAGAAGGTGGTGATGAGGAAGGTGAGGAGGAAGATTAC**TAG**GA**TAA**TATTTTACGAGGAAAAAGCAGCTGTATGTCTAATCTCGTCTCATTCATGTGTGTTTGTCTAGACGTGTACAATCATCATTATTATCCATGTCAGTGATCTGTGAATTGACTCTCCATTCTGT

>PdTUA7

TGCTCTGGAAAAGGATTATGAGGAAGTTGGAGCAGAGTCCGCTGAAGGGGAAGACGATGATGGAGAGGAATACATGTAATGCCAGGTGTTTTTTGCGGGGAAGTTGTTCTGCCAATGGGCTCATTGGTTTGTAACAAGGGCATGGATAGGACTAGTTGTTGTTGTTAGGTTTTACAGTAACTGGATGGACAAGAATGATT

>PdTUA8

TGCCCTTGAAAAAGACTACGAGGAAGTTGGAGCAGAAGGTGGTGATGACGAGGGTGAGGACGAAGATTACTGAGATAATATTTTGCGAGGAAACAGCAGTTGTATGTCTATCTCTTATCATTCATGTGCGTTTGTCTAGAGGTGTACTATCATCATTATTTCCATGTCAGTGATCTGTGAATTGTCTGATCTGTGAATTG

>PtaTUA1_alelle1

TGCCCTGGAGAAGGATTATGAGGAGGTTGGGGCTGAATCTCCTGATGGAGAGGATGGTGATGAAGGAGATGAGTACTGATAAATAGGGAGTGTACTTCCTTTTGTGTTTTTGTTTGGCTGCTATGAGTTTGCTTAGACCATGTCTGTTGGTACGTGTGATCTTTGGTATTGTAATAAAAAAAAATACAAGGATTGGAGGC

>PtaTUA1_alelle2

TGCCCTGGAGAAGGATTATGAGGAGGTTGGGGCTGAATCTCCTGATGGAGAGGATGGTGATGAAGGAGATGAGTACTGATAATAGGGAATGTACTTCCTTTTGTGTTTTTGTTTGGCTGCTATGAGTTTTTGCTTAGACCATGTCTGTTGGTACGTGTGATCTTTGGTATTGTAATAAAAAAAAATACAAGGATTGGAGG

>PtaTUA2

CGCCCTTGAGAAGGATTACGAGGAAGTTGGTGCTGAAGGTGTAGATGATGAAGAAGACAATGAAGATTACGAATAGGTTATTACCACCAGCAATCGAGAAGGACAGTGAGGGTTTGGCCACCAACCCTCCCCTTCTTGCTTCGCAGCTGTTACGTTTGTGATTTGTGGCACTGCTATCCTTCAGTAAAACAGTTAAAAAA

>PtaTUA3

TGCACTCGAGAAGGATTATGAAGAGGTGGGTGCGGAATCAGCCGAGGGCGACGATGAAGACGGCGAGGAGTACATGTGAGGTGCTGCATTACCTCTTTCTCCAATGAAGTGCTTGACACTTCGATACGAGTTGTTGTTTGGCAGTGCCATGTGTCCTGTTCCAAAGGTGTTGAAGGAACCCTTTTATATTTAAAGCTAAT

>PtaTUA4

CGCCCTTGAGAAGGATTACGAGGAAGTTGGTGCTGAAGGTGTAGATGATGAAGAGGAAGGCGATGACTATCAATAAGTTATTGCCACTTGCAATAGAATTCATCTGGGAAATGGTGAAGCGTGTGAGGATTTGGCCACCAACCCGCCCGTCTTACGTTGCCGTTGTTATGTTTGTGATGTGTCGCACCACTATCCTTCTT

>PtaTUA5.2

TGCCCTTGAGAAGGATTATGAGGAGGTTGGTGCTGAATCAGCTGAGGGTGATGATGATGATGGTGACGAGTACATGTGAAGTGCTTCGTAATGGGTGTAGCCTAATATGATGCGTGTTTCTGTTTGGCATTGCCATATGTTGTATTTTATTTTTTAATGAAAATCGGCATGTAATTGCACCATTTGAAGCTTTTATTGTT

>Potri.013G056800|TUA6

TGCTCTTGAGAAAGACTATGAGGAAGTCGGAGCAGAAGGTGGTGATGAGGAAGGTGAGGAGGAAGATTAC**TAG**GA**TAA**TATTTTACGAGGAAAAAGCAGCTGTATGTCTAATCTCGTCTCATTCATGTGTGTTTGTCTAGACGTGTACAATCATCATTATTATCCATGTCAGTGATCTGTGAATTGACTCTCCATTCTGT

>PtaTUA7_allel1

TGCTCTGGAAAAGGATTATGAGGAAGTTGGGGCAGAGTCCGCTGAAGGGGAAGACGATGAAGGAGAGGAATACATGTAATGCCAGGTGTTCTTTGCGGGGAAGTTGTTCTGCCAATGGGCTCATTGGTTTGTAACAAGGGCATGGATAGGACTAGTTGTTGTTGTTAGGTTGTAGGTAACTGGATGGACAAGAATGATTT

>PtaTUA7_allele2

TGCTCTGGAAAAGGATTATGAGGAAGTTGGGGCAGAGTCCGCTGAAGGGGAAGACGATGAAGGAGAGGAATACATGTAATGCCAGGTGTTCTTTGCGGGGAAGTTGTTCTGCCAATGGGCTCATTGGTTTGTAACAAGGGCATGGATAGGACTAGTTGTTGTTAGGTTGTAGGTAACTGGATGAACAAGAATGATTTAGA

>PtaTUA8

TGCTCTTGAGAAAGACTACGAGGAAGTCGGAGCAGAAGGTGGTGATGATGAGGGTGAGGACGAAGATTACTGAGATAATATTTTGTGAGGAAATAGCAGTTGTATGTCTATCTCTTATCATTCGTGTGCGTTTGTCTAGAGGTGTACTATCATCATTATTTCCATGTCAGTGATCTGTGAATTGTCTGTCCATTATGTAT

>PdTUB1

GAGAGTAACATGAATGATCTGGTTTCCGAGTATCAGCAATATCAAGATGCAACCGCTGATGAGGAAGGAGAATATGAGGATGAGGAAGAAGGAGAATACCAGGGAGATTATCAGTAGATTTTCTTTTCAACCTCAACTTCCAGTTGCTGGTTTCCTTTCGTCGTTTTGCTATCAATGAATTTGCAGCTAGGGATCAATTA

>PdTUB2

GAGAGTAACATGAATGATCTGGTTTCTGAGTATCAGCAATACCAAGATGCAACAGCTGATGAGGAAGGAGAATACGAGGAGGAGGAGGAAGGAGAGGAATATCAGCAAGACTATCAGTAGATT

>PdTUB3

GAGAGCAACATGAATGATTTGGTCTCAGAGTACCAGCAATACCAGGATGCAACTGCTGATGAAGAAGGCGAGTTTGAAGATGAGGAGGAAGCATACGGGGATGAGGCCTAAAGACATGAATCTGAATCTTTCCTTCGTTTGCTTATAGGATGTATGGATTGACTGATTTACTTTTGTTCTACTTTTTTTCTGTTACATGC

>PdTUB4

GAGAGCAACATGAATGATTTGGTCTCAGAGTACCAGCAATACCAGGACGCAACTGCTGATGAGGAAGGCGAGTATGAAGATGAGGAAGCGTACCAGGACGAGGATTAAAGATGAGAGATATCTGTCTTTCCTTCGTTTGCTTATTGGATATACGGGTGTCTGGTTTACTTCTGTTGTATGTTTTTTTTTCTGTCATCTGC

>PdTUB5

GAGAGCAACATGAATGATTTGGTTTCTGAGTATCAGCAGTACCAAGATGCCACAGCCGATGAGGACTATGAGGATGAAGAGGAGGAACTCCATGACATGTGATCTTGATCCATGTTATCATGTTGAATGAAAATGCTTGGAGTTGGGTTGTGATTGAGGTAGTAGACGTTGAAAGAAATGCAAGCCAACATTCTGCGGTC

>Potri.016G109300|TUB6

GAGAGCAACATGAATGATTTGGTGTCCGAGTATCAGCAGTACCAAGATGCCACAACTTACGAGGACTGTGAGGATGAAGAGGAACTCCATGACATG**TGA**TCTTGTTCTATGTTGCTATGCTGAATGAGAATGCTTGGAATTGGGTTGTGAATGAGGTAGTGGATGTTGAGAGAAATGCAAGCCAACGTTCTGTAGTCAGT

>PdTUB7

GAGAGCAACATGAATGATCTCGTGGCAGAGTACCAGCAATACCAGGATGCAACTGCTGATGACGAGGAGTACGAGGAGGAGGAAGAAGAGGAAATTGGTGCTTAAGATAGCTTGAAATTCTTGTCGGCATGTCTCAAGTTTGTATTTTATTTGCTCTATTTAAGTGAGTG

>PdTUB8

GAGAGTAACATGAACGATCTGGTTGCTGAGTATCAGCAATACCAGGATGCAACTATCGATGAAGAGGAGTACGAGGAGGAAGAGGAAGAGGAACATGATACTTAAGATAGCTTGAAATTCTTTTCACTATGTCTGAAGTATGTATTTCCCTGGCTCTATTTAAGTGAGTGATGTGTTTCTCATTTGGGGGTCAAGCTAGA

>PdTUB9

GAAAGTAACATGAACGATTTGGTTTCTGAATATCAACAATATCAAGATGCCGTAGCCGATAATGAAGGGGAGTATGATGAAGAAGAGCCTATGGAGAACTAAGGAGAATTTGATCTGGTTATTTGCCTTTGGCTATGGCTTCAAGGACGAGTTGTTTCTGGTGGAGTTATTTATGTTACTGTATGGAGGTCAATATTTGA

>PdTUB10

GAAAGTAACATGAATGATTTGGTTTCTGAGTATCAACAATATCAAGATGCCGCTGCGGATAATGACGACGAGTATGATGAAGAAGAGATAGTGGAGAACTAAAGGGCTATTTGACCAGGTTGTTGTTTGGCTATGGTTATGGCTGTAATAAGGATGTTTTTTTTTTCCTTTTCTTTTTTAACGGAGTTAATTTATGTTTA

>Potri.012G047600|TUB11

GAGAGCAATATGAATGATCTTGTGGCCGAGTACCAACAGTACCAGGATGCCACTGCTGAAGAAGAAATTGAGTATGAGGAGGATGATGGAGTTGAAAAC**TGA**AGGTGTTCAGTTCAGTTATTTTGCTGCTTCATATGTTACGTGCTGCAGTAAATCGATAGGTTTATGTTTTCTCATTTAAAGTCTTATGACGATGACGG

>Potri.015G038800|TUB12

GAGAGCAACATGAATGATCTTGTTGCTGAGTATCAACAGTACCAAGATGCCACTACTGAAGAAGATATTGAGTATGAGGAGGAGGATGGAGTCGAAAAC**TGA**AGGTGTTCAGCTATTTTGCTGCTTCGTATGCTGTGTGCTGCAGTAAATCGATAGGTTTTGTTTTTATATTTCAAATGTTGTGAAGACAGTGATGAATG

>PdTUB13

GAAAGCAACATGAATGACCTCGTGTCTGAATATCAGCAATACCAGGATGCAACTGCTGAGGATGATATTGACTACGAGGATGAGGAGGAAGAAGAGGCTGCTGAGATGTAAAGAAGTGTGAGCGGTCCGGCGACCGATTTTGCAGTCTTGCTGCATAAATGTGTGTACTCTTTTA

>Potri.009G067100|TUB14

GAGAGCAACATGAATGACCTCGTGTCTGAATATCAGCAGTACCAGGATGCAACAGCTGATGAAGAAGTTGATTATGAGGATGAGGAGGAAGAAGAGGCTGAGATG**TGA**TGAGGTGTAAACGGTCCTGCTACTGTTTTTTTTTTTGCAGTTTATGCTGCGTTAATTTGTACTCTTCAAATTTGTCATGTGTTGAATTTTCT

>PdTUB15 Run5.c23_g1_i4

GAAAGCAACATGAATGATCTCGTGTCTGAATATCAGCAATACCAGGATGCAACCGTTGATGAAGAACTTGAGTACGAGGATGAGGAGGAGGAAGAGGCTGCCTAGATGTAATGAGTTGTGCAAGGTCTATCTTCTGCTGCTGCTATTTTTCATACATTTTTACCGCTTTAAGTTCCGCGTGGTGATTTTCACTGTCTGAG

>PdTUB16

GAGAGCAACATGAATGACCTCGTTTCTGAATATCAGCAGTACCAGGACGCAACAGCTGATGAAGAAGTTGATTATGAGGACGAGGAGGAAGAAGATGCTGCCGGGATGTAATTATTTGTTCAAAGTCTTTCTGCTACTTGCTTTTACAGCGTATTCTGCATGAATATGTTTTACTGCTTTGAAATGTGCGTGGTGAATTT

>PdTUB17

GAGAGTAACATGAATGATCTTGTGTCCGAGTATCAGCAGTATCAAGATGCCACTGCTGATGAGGAGGGTGAGTACGAAGACGAGGAAGATGGCCAGTACGCTGAGCAGATGTGAGATGTTTAATTTTGCACAGTAATGTCGACAGGTATATGTTTGCTCCATTATATATATTATTGCGGAATCCTATATTTGCTACATTC

>PdTUB18

GAGAGCAACATGAATGATCTCGTGTCTGAGTACCAGCAGTACCAGGATGCCACTGCTGACGAGGAGGGTGAGTACGATGACGAGGAAGAAGAGGAGGGTCAGTATGCGGAGCAGATGTGAAGGAGTGTCACAGCCAAGCATGAACAGTAATATCCGATTCCTATCGACGCTTCACTATGGTATATGAAGCTCGCTTAAGT

>PdTUB19

GAGAGCAACATGAATGATCTCGTGGCAGAGTACCAGCAATACCAGGATGCAACAATTGAGGAAGATGGTGAATATGAGGAGGAAGGTGAAGAGAACTATGATGCCTAGGGAGCATATGATCTGTGCTGTGCTATTTCATCAAAGCTGTAATGGGGTGTTTCATCATCTGCCAACCTTGCTGGGTATCTAATACTATATTC

>PdTUB20

GAGAGCAACATGAACGATCTAGTGGCAGAGTACCAGCAGTACCAGGATGCAACAGTTGAGGAAGATGGTGAATACGAGGAGGAAGGTGAGGAGAACTATGATGACTGAGAAGCATATGAATTGTGGCTATTTCCTTCTATTGATGTTTCTCAATAGCGCTGCTGGCTGTCACTATATTCTTTGTTGTTTTCGACTTCTCA

>PtaTUB1

GAGAGTAACATGAATGATCTGGTTTCCGAGTATCAGCAATATCAAGATGCAACCGCTGATGAGGAAGGAGAATATGAGGACGAGGAAGAAGGAGAATACCAGGGAGATTATCAGTAGATTTTCTTTTCAACCTCAACTTCCAGTTGCTGGTTTCTTTTCGTCTTTTTGCTATCAATGAATTTGCAGCTAGGGATCAATTA

>PtaTUB2

GAGAGTAACATGAATGATCTGGTTTCTGAGTATCAACAATACCAAGATGCAACAGCTGATGAGGAAGGAGAATACGAGGAGGAGGAGGAAGGAGATGAATATCAGCAAGACTATCAGTAGATTTTCTCTCTTTTGAACCTCAACTTCCAGTTGCTGGTTCTATTCTTCTTTAGTATGTAGGGTTTTAAAATGAATCTGCA

>PtaTUB3

GAGAGCAACATGAATGATTTGGTCTCAGAGTACCAGCAATACCAGGATGCAACTGCTGATGAGGAAGGCGAGTTTGAAGATGAGGAGGAAGCATACGGGGATGAGGCCTAAAGACATGAAATCTGAATCTTTCCTTCGTTTGCTTATAGGATGTATGGATTGACTGATTTACTTTTGTTCTACTTTTTTTCTGTTACATG

>PtaTUB4

GAGAGCAACATGAATGATTTGGTCTCAGAGTACCAGCAATACCAGGACGCAACTGCTGACGAGGAAGGCGAGTATGAAGACGAGGAAGCATACCAGGACGAGGATTAAAGATGAGAGATATCTGTCTTTCCTTCGTTTGCTTATAGGATATACGGGTGTATGGTTTCCTTCTGTTGTATGTTTTTTATCTGTCATGTGCT

>PtaTUB5

GAGAGCAACATGAATGATTTGGTTTCTGAGTATCAGCAGTACCAAGATGCCACAGCCGATGAGGACTATGAGGATGAAGAGGAGGAACTCCATGACATGTGATCTTGATCCATGTTATCATGTTGAATGAAAATGCTTGGAGTTGGGTTGTGATTGAGGTAGTAGACGTTGAAAGAAATGCAAGCCAACATTCTGCGGTC

>Potri.016G109300|TUB6

GAGAGCAACATGAATGATTTGGTGTCCGAGTATCAGCAGTACCAAGATGCCACAACTTACGAGGACTGTGAGGATGAAGAGGAACTCCATGACATG**TGA**TCTTGTTCTATGTTGCTATGCTGAATGAGAATGCTTGGAATTGGGTTGTGAATGAGGTAGTGGATGTTGAGAGAAATGCAAGCCAACGTTCTGTAGTCAGT

>Potri.001G464400|TUB7

GAGAGTAACATGAATGATCTGGTAGCTGAGTACCAGCAATACCAGGATGCAACTGCTGATGACGAGGAGTACGAGGAGGAGGAAGAAGAGGAAATTGGTGCT**TAA**AATTCTTGTCGGCATGTCTGAAGTTTGTATTTTATTTGCTCTATTTAAGTGAGTGATTATGTTTCTGTTGTTGGGGGGTCAAATTAGAGTGACCT

>PtaTUB8

GAGAGTAACATGAACGATCTGGTTGCTGAGTATCAGCAATACCAGGATGCAACTATCGATGAAGAGGAGTACGAGGAGGAAGAGGAAGAGGAACATGATACTTAAGATAGCTTGAAATTCTTTTCACTATGTCTGAAGTATGTATTTCCCTGGCTCTATTTAAGTGAGTGATGTGTTTCTCATTTGGGGGTCAAGCTAGA

>PtaTUB9

GAAAGTAACATGAACGATTTGGTTTCTGAATATCAACAATATCAAGATGCCGCAGCCGATAATGAGGGGGAGTATGATGAAGAAGAGCCTATGGAGAACTAAGGAGAATTTGATCTGGTTATTTTCCTATGGCCATGGCTTCAAGGATGAGTTGTTTCTGGTGGAGTTATTTATGTTACAGTATGGAGGTCAATATTTGA

>PtaTUB10

GAAAGTAACATGAATGATTTGGTTTCCGAGTATCAACAATATCAAGACGCCGCTGCGGATAATGACGACGAGTATGATGAAGAAGAGGCAATGGAGAACTGAAGGGGCTATTTGATCAGGTTGTTGTTTGGCTATGGTTATGGCTGTAATAATGATGTTTTTTTTTTGTACGGAGTTAAT

>Potri.012G047600|TUB11

GAGAGCAATATGAATGATCTTGTGGCCGAGTACCAACAGTACCAGGATGCCACTGCTGAAGAAGAAATTGAGTATGAGGAGGATGATGGAGTTGAAAAC**TGA**AGGTGTTCAGTTCAGTTATTTTGCTGCTTCATATGTTACGTGCTGCAGTAAATCGATAGGTTTATGTTTTCTCATTTAAAGTCTTATGACGATGACGG

>Potri.015G038800|TUB12

GAGAGCAACATGAATGATCTTGTTGCTGAGTATCAACAGTACCAAGATGCCACTACTGAAGAAGATATTGAGTATGAGGAGGAGGATGGAGTCGAAAAC**TGA**AGGTGTTCAGCTATTTTGCTGCTTCGTATGCTGTGTGCTGCAGTAAATCGATAGGTTTTGTTTTTATATTTCAAATGTTGTGAAGACAGTGATGAATG

>PtaTUB13

GAAAGCAACATGAATGACCTCGTGTCTGAATATCAGCAATACCAGGATGCAACTGCTGAGGATGATATTGACTACGAGGATGAGGAGGAAGAAGAGGCTGCTGAGATGTAAAGAAGTGTGAGCGGTCCGGCGACCGATTTTGCAGTCTTGCTGCATAAATGTGTGTACTCTTTTA

>Potri.009G067100|TUB14

GAGAGCAACATGAATGACCTCGTGTCTGAATATCAGCAGTACCAGGATGCAACAGCTGATGAAGAAGTTGATTATGAGGATGAGGAGGAAGAAGAGGCTGAGATG**TGA**TGAGGTGTAAACGGTCCTGCTACTGTTTTTTTTTTTGCAGTTTATGCTGCGTTAATTTGTACTCTTCAAATTTGTCATGTGTTGAATTTTCT

>PtaTUB15

GAAAGCAACATGAATGACCTCGTGTCTGAATATCAGCAGTACCAGGATGCAACCGTTGATGAAGAACTTGAGTACGAGGATGAGGAGGAGGAAGAGGCTGCCTAGATGTAATGAGTTGTGCAAGGTCTATCTTCTTCTGCTGCTGCTTTTTTTCATACATTTTTACCGCTTTAAGTTCCGGGTTGGAAAATTGCCTATAT

>PtaTUB16

GAGAGCAACATGAATGACCTCGTGTCTGAATATCAGCAGTACCAGGATGCAACAGCCGAAGAAGAGGTTGATTATGAGGACGAGGAGGAAGAAGATGCTGCCGGGATGTAATTATTTGTGCAAAGTCTATCTGCTACTTGCTTTTACAGCGTATTCTGCATGAATATGTTTACTGCTTTCAAATGTGCGTGGTGAATTTC

>PtaTUB17

GAGAGTAACATGAATGATCTTGTGTCCGAGTATCAGCAGTATCAAGATGCCACTGCTGATGAGGAGGGTGAGTACGAAGACGAGGAAGATGGCCAGTACGCGGAGCAGATGTGAAATGTTTAATTTTGCACAGTAATGTTGACAGGTATATATTTGCTCCATTATATATATTATTGAGGAATCTTATATTTGCTACATTC

>PtaTUB18

GAGAGCAACATGAATGATCTTGTTTCCGAGTACCAGCAGTACCAAGATGCCACCGCTGATGAGGAGGGTGAGTATGATGACGAGGAGGAAGAGGAGGGTCAGTATGCAGAGTAGATGTGAAGGTGTGTCACAACCAAGCATGAACAATAATATCCGATGCGTATCGATGCTTCACTATGGTATATGAAGCTCGCGTAAGT

>PtaTUB19

GAGAGCAACATGAACGATCTCGTGGCAGAGTACCAGCAATACCAGGATGCAACAATTGAGGAAGATGGTGAATATGAGGAGGAAGGTGAAGAGAACTACGATGCCTAGGGAGCACATGATCTGTGCTGTGCTCTTTCATCAAAGCTGTTCTGTAATGGGGTTTCATCATCTGCCAACCTTGCTGGGTATCTAATACTATA

>PtaTUB20

GAGAGCAACATGAACGATCTAGTGGCAGAGTACCAGCAGTACCAGGATGCAACAGTTGAGGAAGATGGTGAATACGAGGTGGAAGGTGAGGAGAACTATGATGACTGAGAAGCATATGAATTGTGGCTATCTCCTTAACGCTGGAATGGGG

**Supplementary Data 2.** Sequence alignment of the predicted *P. trichocarpa* *TUA5* splice variants with the *P. deltoides* and *P. tremula* × *alba* transcripts obtained from *de novo* assembly. Predicted start and stop codons are shown in red.

P.deltoidesTUA5 CTTCGTTTCTTATCTCCTTCTTCAACAATCAGATCTATCTTTGTTGTTTCTTTAATCAGCGAAA**ATG**AGAGAGTGCATTTCGATCCACATTGGTCAAGCCGGTAT

P.tremulaxalbaTUA5 CTTCGCTCCTAATCTCCTTCTTCAACAATCAGATCTATCTTTGTTGTTTCTTTAATCAGCGAAA**ATG**AGAGAGTGCATTTCGATCCACATTGGTCAAGCCGGTAT

Potri.009G085100.1 CTTCGTTCCTAATCTCCTTCTTCAACAATCAGATCTATCTTTGTTGTTTCTTTAATCAGCGAAA**ATG**AGAGAGTGCATTTCGATCCACATTGGTCAAGCCGGTAT

Potri.009G085100.2 CTTCGTTCCTAATCTCCTTCTTCAACAATCAGATCTATCTTTGTTGTTTCTTTAATCAGCGAAA**ATG**AGAGAGTGCATTTCGATCCACATTGGTCAAGCCGGTAT

***** * ** **********************************************************************************************

P.deltoidesTUA5 TCAAGTCGGAAATGCTTGCTGGGAACTCTACTGCCTCGAGCATGGCATCCAGCCTGATGGTCAGATGCCAAGTGACAAGACTGTTGGTGGAGGGGATGATGCCTT

P.tremulaxalbaTUA5 TCAAGTCGGAAATGCCTGCTGGGAACTCTACTGCCTCGAGCATGGCATCCAGCCTGATGGTCAGATGCCGAGTGACAAGACTGTTGGTGGAGGGGACGATGCCTT

Potri.009G085100.1 TCAAGTCGGAAATGCCTGCTGGGAACTCTACTGCCTCGAGCATGGCATCCAGCCTGATGGTCAGATGCCAAGTGACAAGACTGTTGGTGGAGGGGATGATGCCTT

Potri.009G085100.2 TCAAGTCGGAAATGCCTGCTGGGAACTCTACTGCCTCGAGCATGGCATCCAGCCTGATGGTCAGATGCCAAGTGACAAGACTGTTGGTGGAGGGGATGATGCCTT

*************** ***************************************************** ************************** ********

P.deltoidesTUA5 TAACACCTTTTTCAGTGAAACTGGTGCCGGGAAGCACGTCCCTCGTGCTGTCTTTGTAGATCTTGAGCCCACTGTCATTGATGAAGTCAGGACTGGAACTTACCG

P.tremulaxalbaTUA5 TAACACCTTTTTCAGTGAAACTGGTGCCGGGAAGCACGTCCCTCGTGCTGTCTTTGTAGATCTTGAGCCCACTGTCATTGATGAAGTCAGGACTGGAACTTACCG

Potri.009G085100.1 TAACACCTTTTTCAGTGAAACTGGTGCCGGGAAGCACGTCCCTCGTGCTGTCTTTGTAGATCTTGAGCCCACTGTCATTGATGAAGTCAGGACTGGAACTTACCG

Potri.009G085100.2 TAACACCTTTTTCAGTGAAACTGGTGCCGGGAAGCACGTCCCTCGTGCTGTCTTTGTAGATCTTGAGCCCACTGTCATTGATGAAGTCAGGACTGGAACTTACCG

*********************************************************************************************************

P.deltoidesTUA5 CCAACTTTTCCACCCTGAACAACTTATTAGCGGCAAGGAAGATGCTGCCAACAACTTTGCCCGTGGACACTATACCATTGGCAAGGAAATTGTTGACCTGTGCTT

P.tremulaxalbaTUA5 CCAACTTTTCCACCCCGAACAACTTATTAGCGGCAAGGAGGATGCTGCCAACAACTTCGCCCGTGGACACTATACCATTGGCAAGGAAATTGTTGACCTGTGCTT

Potri.009G085100.1 CCAACTTTTCCACCCTGAACAACTTATTAGCGGCAAGGAGGATGCTGCCAACAACTTTGCCCGTGGACACTATACCATTGGCAAGGAAATTGTTGACCTGTGCTT

Potri.009G085100.2 CCAACTTTTCCACCCTGAACAACTTATTAGCGGCAAGGAGGATGCTGCCAACAACTTTGCCCGTGGACACTATACCATTGGCAAGGAAATTGTTGACCTGTGCTT

*************** *********************** ***************** ***********************************************

P.deltoidesTUA5 GGACCGTATCCGAAAGCTAGCTGACAACTGTACTGGTCTGCAAGGCTTCCTTGTATTCAATGCTGTTGGTGGTGGTACTGGATCTGGTCTTGGGTCCCTTCTCTT

P.tremulaxalbaTUA5 GGACCGTATCCGAAAACTCGCTGACAACTGTACTGGTCTGCAAGGCTTCCTTGTATTCAATGCTGTTGGTGGTGGTACTGGATCTGGTCTTGGGTCCCTTCTCTT

Potri.009G085100.1 GGACCGTATCCGAAAGCTCGCTGACAACTGTACTGGTCTGCAAGGCTTCCTAGTATTCAATGCTGTTGGTGGTGGTACTGGATCTGGTCTTGGGTCCCTTCTCTT

Potri.009G085100.2 GGACCGTATCCGAAAGCTCGCTGACAACTGTACTGGTCTGCAAGGCTTCCTAGTATTCAATGCTGTTGGTGGTGGTACTGGATCTGGTCTTGGGTCCCTTCTCTT

*************** ** ******************************** *****************************************************

P.deltoidesTUA5 GGAGCGCTTGTCTGTTGACTATGGAAAGAAATCCAAGTTGGGTTTCACCGTGTATCCATCTCCTCAGGTGTCCACATCTGTTGTTGAGCCCTACAACAGTGTTCT

P.tremulaxalbaTUA5 GGAGCGCTTGTCTGTTGACTATGGAAAGAAATCCAAGTTGGGTTTCACCGTGTATCCATCTCCTCAGGTGTCCACATCTGTTGTTGAGCCCTACAACAGTGTTCT

Potri.009G085100.1 GGAGCGTTTGTCTGTTGACTATGGAAAGAAATCCAAGTTGGGTTTCACTGTGTATCCATCTCCTCAGGTGTCCACATCTGTTGTTGAGCCCTACAACAGTGTTCT

Potri.009G085100.2 GGAGCGTTTGTCTGTTGACTATGGAAAGAAATCCAAGTTGGGTTTCACTGTGTATCCATCTCCTCAGGTGTCCACATCTGTTGTTGAGCCCTACAACAGTGTTCT

****** ***************************************** ********************************************************

P.deltoidesTUA5 CTCAACTCACTCCCTTTTGGAACACACAGATGTGGCTGTGCTTCTTGACAATGAAGCTATCTATGATATCTGCAAGCGTTCTCTTGACATTGAGCGACCCACCTA

P.tremulaxalbaTUA5 CTCAACTCACTCCCTTTTGGAACACACAGATGTGGCTGTGCTTCTTGACAATGAAGCTATCTATGATATCTGCAAGCGCTCTCTTGACATTGAGCGACCCACCTA

Potri.009G085100.1 CTCAACTCACTCCCTTTTGGAACACACAGATGTGGCTGTGCTTCTTGACAATGAAGCTATCTATGATATCTGCAAGCGTTCTCTTGACATTGAGCGACCCACCTA

Potri.009G085100.2 CTCAACTCACTCCCTTTTGGAACACACAGATGTGGCTGTGCTTCTTGACAATGAAGCTATCTATGATATCTGCAAGCGTTCTCTTGACATTGAGCGACCCACCTA

****************************************************************************** **************************

P.deltoidesTUA5 CACCAACCTCAACAGGCTTATCTCTCAGGTCATTTCCTCCTTGACCGCCTCTCTGAGGTTTGATGGTGCTTTGAATGTGGATGTTACTGAATTCCAGACTAACTT

P.tremulaxalbaTUA5 CACCAACCTCAACAGGCTTATCTCTCAGGTCATTTCCTCCTTGACCGCCTCTCTGAGGTTTGATGGTGCTTTGAATGTGGATGTTACTGAATTCCAGACTAACTT

Potri.009G085100.1 CACCAACCTCAACAGGCTTATCTCTCAGGTCATTTCCTCCTTGACCGCCTCTCTGAGGTTTGATGGTGCTTTGAATGTGGATGTTACTGAATTCCAGACTAACTT

Potri.009G085100.2 CACCAACCTCAACAGGCTTATCTCTCAGGTCATTTCCTCCTTGACCGCCTCTCTGAGGTTTGATGGTGCTTTGAATGTGGATGTTACTGAATTCCAGACTAACTT

*********************************************************************************************************

P.deltoidesTUA5 GGTCCCTTACCCAAGAATCCACTTCATGCTTTCCTCCTATGCACCAGTCATCTCTGCTGAGAAAGCCTACCATGAACAACTCTCTGTTGCTGAGATCACTAACAG

P.tremulaxalbaTUA5 GGTCCCTTACCCAAGAATCCACTTCATGCTTTCCTCCTATGCACCAGTCATCTCTGCTGAGAAAGCCTACCATGAACAACTCTCTGTTGCTGAGATCACTAACAG

Potri.009G085100.1 GGTCCCTTACCCAAGAATCCACTTCATGCTTTCCTCCTATGCACCAGTCATCTCTGCTGAGAAAGCCTACCATGAACAACTCTCTGTTGCTGAGATCACTAACAG

Potri.009G085100.2 GGTCCCTTACCCAAGAATCCACTTCATGCTTTCCTCCTATGCACCAGTCATCTCTGCTGAGAAAGCCTACCATGAACAACTCTCTGTTGCTGAGATCACTAACAG

*********************************************************************************************************

P.deltoidesTUA5 TGCTTTTGAGCCTTCATCAATGATGGCCAAATGTGATCCTCGCCATGGAAAGTACATGGCCTGTTGTCTGATGTACCGTGGTGATGTTGTGCCCAAGGATGTTAA

P.tremulaxalbaTUA5 TGCTTTTGAGCCTTCATCAATGATGGCCAAATGTGATCCTCGTCACGGAAAGTACATGGCCTGTTGTCTGATGTACCGTGGTGATGTTGTTCCCAAGGATGTTAA

Potri.009G085100.1 TGCTTTTGAGCCTTCATCAATGATGGCTAAATGTGATCCTCGCCATGGAAAGTACATGGCCTGTTGTCTGATGTACCGTGGTGATGTTGTGCCCAAGGATGTTAA

Potri.009G085100.2 TGCTTTTGAGCCTTCATCAATGATGGCTAAATGTGATCCTCGCCATGGAAAGTACATGGCCTGTTGTCTGATGTACCGTGGTGATGTTGTGCCCAAGGATGTTAA

*************************** ************** ** ******************************************** **************

P.deltoidesTUA5 TGCTGCTGTTGCCACGATCAAGACCAAGCGTACTATTCAGTTTGTTGACTGGTGCCCCACTGGATTCAAGTGTGGTATCAATTACCAGCCACCCACTGTTGTTCC

P.tremulaxalbaTUA5 TGCTGCTGTTGCCACGATCAAGACCAAGCGTACTATTCAGTTTGTTGACTGGTGCCCCACTGGATTCAAGTGTGGTATCAATTACCAGCCACCCACTGTTGTTCC

Potri.009G085100.1 TGCTGCTGTTGCCACGATCAAGACCAAGCGTACTATTCAGTTTGTTGACTGGTGCCCCACTGGATTCAAGTGTGGTATCAATTACCAGCCACCCACTGTTGTTCC

Potri.009G085100.2 TGCTGCTGTTGCCACGATCAAGACCAAGCGTACTATTCAGTTTGTTGACTGGTGCCCCACTGGATTCAAGTGTGGTATCAATTACCAGCCACCCACTGTTGTTCC

*********************************************************************************************************

P.deltoidesTUA5 TGGTGGTGATCTTGCCAAGGTGCAGAGGGCTGTTTGCATGATCTCCAACTCTACCAGTGTTGCTGAGGTGTTCTCTCGCATTGACTCCAAATTTGACCTCATGTA

P.tremulaxalbaTUA5 TGGTGGTGATCTTGCCAAGGTGCAGAGGGCTGTTTGCATGATCTCCAACTCTACCAGTGTTGCTGAGGTGTTCTCTCGCATTGACAGCAAATTTGACCTCATGTA

Potri.009G085100.1 TGGTGGTGATCTTGCCAAGGTGCAGAGGGCTGTTTGCATGATCTCCAATTCTACCAGTGTTGCTGAGGTGTTCTCTCGCATTGACTCCAAATTTGACCTCATGTA

Potri.009G085100.2 TGGTGGTGATCTTGCCAAGGTGCAGAGGGCTGTTTGCATGATCTCCAATTCTACCAGTGTTGCTGAGGTGTTCTCTCGCATTGACTCCAAATTTGACCTCATGTA

************************************************ ************************************ ******************

P.deltoidesTUA5 TGCCAAGCGTGCCTTTGTGCACTGGTATGTTGGTGAGGGCATGGAGGAAGGCGAGTTCTCTGAGGCTCGTGAGGATCTTGCCGCCCTTGAGAAGGATTATGAGGA

P.tremulaxalbaTUA5 CGCCAAGCGTGCCTTTGTGCACTGGTATGTTGGTGAGGGCATGGAGGAAGGCGAGTTCTCGGAGGCTCGTGAGGATCTTGCTGCCCTTGAGAAGGATTATGAGGA

Potri.009G085100.1 TGCCAAGCGTGCCTTCGTGCACTGGTATGTTGGTGAGGGCATGGAGGAAGGCGAGTTCTCTGAGGCTCGTGAGGATCTTGCCGCCCTTGAGAAGGATTATGAGGA

Potri.009G085100.2 TGCCAAGCGTGCCTTCGTGCACTGGTATGTTGGTGAGGGCATGGAGGAAGGCGAGTTCTCTGAGGCTCGTGAGGATCTTGCCGCCCTTGAGAAGGATTATGAGGA

************** ******************************************** ******************** ***********************

P.deltoidesTUA5 GGTCGGTGCTGAATCAGCTGAGGGTGATGATGATGATGGTGACGAGTACATG**TGA**AGTGCTTCGTAGTGGGTGTAGCCCTAATATGATG--TGTATCTGTATGGC

P.tremulaxalbaTUA5 GGTTGGTGCTGAATCAGCTGAGGGTGATGATGATGATGGTGACGAGTACATG**TGA**AGTGCTTCGTAATGGGTGTAG-CCTAATATGATGCGTGTTTCTGTTTGGC

Potri.009G085100.1 GGTTGGTGCTGAATCAGCTGAGGGTGATGATGATGATGGTGACGA--------------------------------CCTAATA**TGA**TG--TGTTTCTGTATGGC

Potri.009G085100.2 GGTTGGTGCTGAATCAGCTGAGGGTGATGATGATGATGGTGACGAGTACATG**TGA**AATGCTTCGTAGTGGGTGTAG-CCTAATATGATG--TGTTTCTGTATGGC

*** ***************************************** ************ *** ***** ****

P.deltoidesTUA5 ATTGCCGTATGTTGTATT-TGTTTTTTAATGAATATCTACATGTAATTGCACCATTCGAAGCTTTTATTGTTTCAACGGTGTGTTTAAAACCTACCTTCGTATGC

P.tremulaxalbaTUA5 ATTGCCATATGTTGTATTTTATTTTTTAATGAAAATCGGCATGTAATTGCACCATTTGAAGCTTTTATTGTTTCGAAGGTGTGTAAAAA-CCTATCTTCGTATGC

Potri.009G085100.1 ATTGCCATATGTTGTATTTTTATTTTTAATGAATATCTACATGTAATTGCACCATTTGAAGCTTTTATTGTTTCAAAGGTGTTTAAAAT--CTACCTGCGTATGC

Potri.009G085100.2 ATTGCCATATGTTGTATTTTTATTTTTAATGAATATCTACATGTAATTGCACCATTTGAAGCTTTTATTGTTTCAAAGGTGTTTAAAAT--CTACCTGCGTATGC

****** *********** * *********** *** ***************** ***************** * ***** * ** *** ** *******

P.deltoidesTUA5 TAAAGCCA----TGTCTACTCTACATGGAATGTGATGGACGACTGTTATTGAAAGTTTTTATTATTATTATTATTATTTATTGGTTGCATTGCCATTAGTTGATC

P.tremulaxalbaTUA5 TAAAGCTACTGTTGTCTACTCTATATGGAATGTGATGGGCGTCTGTTGTTGAAAGATTTTTATTTTATT-------TTTTTCTGTTCCATTGCCATTAGTTGATC

Potri.009G085100.1 TAAAGCCA----TGTCTACTCTACATGGAATGTGATGGACGACTGTTATTGAAAGTTTTTTTTTTTTTT------------TGGTT-------------------

Potri.009G085100.2 TAAAGCCA----TGTCTACTCTACATGGAATGTGATGGACGACTGTTATTGAAAGTTTTTTTTTTTTTT------------TGGTTCCATTGCCATTAGTTGATC

****** * *********** ************** ** ***** ******* **** * ** ** ***
